# Gauging acceptability: the utility of a national attitudes survey toward a trial of pressure garment therapy for burns scar management

**DOI:** 10.1186/1745-6215-16-S2-P21

**Published:** 2015-11-16

**Authors:** Susan Wright, Laura Jones, Naiem Moiemen, Margaret Grant, Jonathan Mathers

**Affiliations:** 1 University of Birmingham, Birmingham, West Midlands, UK; 2 University Hospitals Birmingham, Birmingham, West Midlands, UK

## Background

Trials may be difficult to conduct due to a lack of acceptability of the trial question. For example, trials of existing treatments that are established in routine clinical practice without robust supporting evidence might be undermined by a lack of acceptability or presumed difficulty in recruiting patients. The NIHR HTA commissioned a feasibility study of pressure garment therapy for burns scar management as it was thought a full-scale trial would be difficult to conduct.

## Methods

Pegasus is the resulting commissioned mixed-methods feasibility study which consists of an external pilot trial with process evaluation. To gauge acceptability, in addition to the pilot, we conducted a national survey of attitudes towards a full-scale trial amongst the burns clinical community, supplemented by telephone interviews (n=15).

## Findings

Staff in 27 of 29 (n=223) surveyed burns services responded. Although a majority (67%) were in favour of a trial, this headline figure hides a more complex picture, particularly, in relation to clinical equipoise around the trial question. This has clear implications for the conduct of a national trial. We were able to further explore these survey findings via the telephone interviews and with emerging observations from the pilot trial and process evaluation.

## Discussion

We discuss the utility of conducting a national survey of attitudes in addition to a pilot trial amongst a smaller sample of burns services. We reflect on whether this approach may provide insights over and above those identified from a small scale feasibility pilot.

